# Comparative Experimental Study of Eco-Composite Reinforced Concrete Beams Under Flexural Loading: Sustainability Factors vs. Mechanical Performance

**DOI:** 10.3390/polym18070847

**Published:** 2026-03-31

**Authors:** Youssef Bounjoum, Oumayma Hamlaoui, Youssef Bibridne, Hakan Tozan, Irem Duzdar, Naoufal Bouktib, Noureddine Choab, Mohammed Ait El Fqih

**Affiliations:** 1 Ingénierie des Systèmes Intelligents, Industrielle et Mécanique (LISIME), ENSAM, Hassan II University of Casablanca, Casablanca 20360, Morocco; bounjoumyoussef@yahoo.fr (Y.B.); youssefbibrden@gmail.com (Y.B.); simohammed.aitelfqih@univh2c.ma (M.A.E.F.); 2 College of Engineering and Technology, American University of the Middle East, Egaila 54200, Kuwait; hakan.tozan@aum.edu.kw; 3 Department of Industrial Engineering, Faculty of Engineering, Düzce University, Düzce 81000, Türkiye; iremduzdar@duzce.edu.tr; 4 Laboratoire de Sciences de l’Ingénieur, ESTEM-Casablanca, Casablanca 20060, Morocco; bouktib.naoufal@gmail.com (N.B.); cchoab@gmail.com (N.C.)

**Keywords:** reinforced concrete beams, eco-composites, jute fiber, glass fiber, hybrid FRP, flexural strengthening, sustainability indices

## Abstract

This study is an experimental study on flexural strengthening of reinforced concrete beam where three types of epoxy-bonded jacketing systems are used (glass fiber-reinforced composite (GFRC, S1), jute fiber-reinforced composite (JFRC, S2), and hybrid fiber-reinforced composite (HFRC, S3)) and an unjacketed control beam (S0). All the specimens were subjected to displacement-controlled three-point bending to measure the enhancement of strength, stiffness, and energy absorption using mass-normalized (TPM) and synthetic-content-normalized (TSM) performance indices. Jacketing compared to control also raised the maximum load from 11.80 N to 17.10 N for GFRC (+44.9%), to 14.64 N for JFRC (+24.1%), and to 14.89 N of HFRC (+26.2%). The energy taken up rose from 38.44 J (S0), 152.50 J (S1, +297%), 95.32 J (S2, +148%), and 132.79 J (S3, +245%). Flexural strength was also increased to 56.26 MPa (S1), 43.54 MPa (S2), and 51.38 MPa (S3) and yield strength was raised from 10.43 MPa (S0) to 26.40 MPa (S1), 16.84 Mpa (S2), and 23.05 Mpa (S3). The increase of flexural modulus between S0 (4871.33 MPa) and S1 (12,322.34 MPa), S2 (7862.61 MPa), and S3 (10,759.57 MPa) showed the enhancement of the stiffness. Mass-normalized performance showed great overall efficiency in the case of GFRC and HFRC, with TPM = 3.70 and 3.60 J/kg, respectively, and synthetic-content efficiency was higher in the case of JFRC, with TSM = 9.66 J/kg, which is the advantage of low-synthetic reinforcement in energy-based performance. In general, the suggested jacketing systems have a great influence on flexural responsiveness and power absorption, whereby GFRC and JFRC offer maximum capacity and stiffness, respectively, and the greatest efficiency per unit synthetic material, respectively. In terms of novelty, the paper is one of the first to measure the sustainability-based performance of an epoxy-bonded GFRC, HFRC, and bio-based JFRC jacketing, comparing the results in terms of synthetic-content efficiency (TSM) and mass-normalized indices, which reflect the energy absorption benefits per unit of synthetic material.

## 1. Introduction

The rehabilitation of degrading concrete structures is progressively becoming geared towards the goals set in sustainable development, especially in the construction industry. The traditional reinforcement techniques involving the use of steel or synthetic fiber-reinforced polymers (FRPs) have proven to be extremely strong and durable, but their use has posed a considerable burden on the environment because these materials would be involved in energy-intensive manufacturing and recycling. To address these issues, composites reinforced with natural fibers (NFRCs) have received increased popularity in structural use, due to renewability, biodegradability and good strength-to-weight ratio [1,2,3]. Eco-composite natural fibers including jute, flax, and hemp have been extensively researched in structural elements. The fibers have promise in minimizing the environmental impact of civil infrastructure without compromising mechanical performance when well-incorporated within polymeric matrices [4]. However, inconsistency of natural fibers’ quality, moisture sensitivity, and poor performance under harsh environmental conditions make their sole application in high-performance buildings a limitation [5]. These issues has seen the introduction of hybrid composite systems, which are a combination of natural and synthetic fibers (glass or carbon). These hybrid fiber-reinforced composites (HFRCs) have been proven to provide the required mechanical results comparable to traditional FRPs and are partly sustainable due to the use of natural fibers [5,6,7]. These systems have been reported to be beneficial with regards to improved energy absorption, flexural strength, and cracking and delamination resistance under flexural loading. Although many studies have already been conducted on synthetic FRPs and their reinforcement behavior in beams, columns, and slabs, there are not much comparative experimental studies that have been carried out on natural, synthetic, and hybrid composites in flexural beam strengthening. Additionally, the current body of knowledge has given much emphasis on tensile and shear applications, thus creating a gap in the research of flexural retrofitting using eco-composites. Moreover, the recent studies indicated that numerical simulation and experimentation are valuable to comprehend the pattern of damage and the stress distribution in reinforced concrete. A new work by Bounjoum et al. (2024) on CFRP reinforcements demonstrated the effectiveness of combining experimental and simulation studies to evaluate failure response and bond quality [8]. The authors have recently studied the mechanical behavior of hybrid composites of jute and glass fiber in the hybrid fiber-reinforced polymers and observed the mechanical behavior under different post-curing conditions, with emphasis on tensile strength, stiffness, and failure mode of the composites [9]. The study involved both experimental analysis and numerical analyses performed with Abaqus to investigate how temperature-sensitive polymer matrix behavior would affect load transfer and bonding at the interface. Based on these results, the current research finds that the structural applications of the hybrid composites are further extended to retrofitting concrete beams, in which the hybrids are tested in flexural structures. This is with an intent to evaluate their load-bearing capacity, energy absorption, and applicability in the use of sustainable external reinforcements in the context of hybrid natural–synthetic systems, which are relevant in material sciences as well as structural engineering. This research will seek to fill these gaps by exploring and comparing flexural behavior of concrete beams reinforced externally with glass fiber-reinforced composites (GFRC), jute fiber-reinforced composites (JFRC), and their hybrid (HFRC) counterparts. The experimental program determines flexural strength, energy absorption, stiffness, and failure modes to determine the possibility of using these eco-composite systems in structural use. Although the use of sustainable construction materials has increased in popularity, the structural performance of natural and hybrid fiber-reinforced polymer (FRP) composites as external reinforcement of concrete beams is under-researched. The literature available so far has mainly concentrated on the individual effect of synthetic fibers for example the synthetic fiber-reinforced composite, GFRP or natural fibers, for example the jute or flax fibers, in most cases in isolation and either under tensile or impact loading. Nevertheless, the comparative flexural testing in natural (JFRC), synthetic (GFRC), and hybrid (HFRC) composite jackets used on concrete beams is not numerous, particularly with the perspective of quantitative energy dissipation investigation, the way of failure observation, and systematic characterization of force–displacement behavior. Moreover, there is absence of comprehensive evaluation to determine the adverse effects of such eco-composites simultaneously in a single experimental system. The originality of the work is that it has been a comparative assessment of three eco-composites systems on the same structural testing arrangement, which has given a fresh insight into their comparative effectiveness, toughening, and flexural capacity. This way, the study will add workable standards to incorporate the utilization of hybrid fiber composites in green retrofitting practices in structural engineering. It is increasingly being seen as an option to strengthen RC elements using sustainable reinforcement materials (e.g., natural-fiber or hybrid composite jackets). This method can enhance structural performance and minimize ecologically unsustainable methods that are heavily based on high-carbon, entirely synthetic reinforcements. Thermodynamically, durability and long-term efficiency are based on the role of heat and moisture in driving transport, aging, and evolution of damage in the composite system (e.g., thermal expansion mismatch, moisture sorption, interfacial degradation), which directly influences the quality of bonds and energy dissipation during loading [10,11]. It is also critical to investigate the microstructure (fiber–matrix adhesion, porosity, the quality of the interfacial transition zone, crack-bridging traces, and damage patterns) to understand why various jackets have various reactions to stiffness, strength, ductility, and energy absorption, as well as to understand which failure mechanisms govern such a process (e.g., debonding, fiber pull-out, matrix cracking) [12,13,14,15]. Additionally, the research on reinforcement setup and efficiency (type of fibers, layer/ratio, thickness, and bonding quality) helps to create optimized retrofitting designs to make maximum load transfer and post-peak resistance, as well as to create damage-tolerant strengthening methods based on improved sustainability. This type of multiscale insight also has direct application to manufacturability, as it facilitates definition of process-windows, prediction of sensitivity to processing parameters, and reduction of trial and error by relating processing to microstructure and properties that will determine part quality, which, in turn, enhance repeatability and scalability in production [16,17,18,19,20].

## 2. Materials and Methods

### 2.1. Specimen Preparation

**Figure 1:**
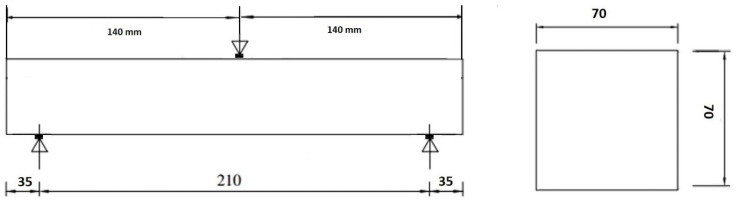
Specimen dimensions.

Four prismatic concrete beam specimens were constructed as prismatic beam with the cross-section of 70 mm × 70 mm × 280 mm, as illustrated in Figure 1. The concrete mix was a normal aggregate mix with compressive strength equal to 30 MPa. Following the general instructions of EN 12390-2:2019 [21] regarding the specimen curing conditions, the beams were demolded after 24 h and left to cure in water at ambient temperature for 28 days.

This operation has been repeated 3 times for each configuration to obtain a total of 12 specimens with different layer configurations as described by Section 2.2.

Three of the twelve specimens had no reinforcement (control specimen S0), and the rest were externally strengthened by composite jacketing with hand lay-up methods. Epoxy resin was used to glue the reinforcement composites on the entire outer surface of the beams.

The beams were marked in the following way: The reinforcement layers were all placed in a bidirectional woven fabric form to increase the isotropic strength behavior. The fibers were arranged in parallel and perpendicular to the beam axis to withstand flexural as well as shear stresses, similar to approaches employed in EN 1504-4:2004 [22] in structural strengthening using bonded composites. For the concrete, CPJ 45 cement was used to produce a B30 normal-weight concrete. The choice of cement grade (minimum compressive strength of 42.5 MPa) was to secure a stable post-hardening performance and high-quality binder phase, and the desired concrete grade aligned with the characteristic strength of 30 Mpa. Mixing and curing were performed with potable water; its compliance with CSA A23.1, Clause 4.2.2, and ASTM C94, as well as the chemical analysis, ensured its adherence to the two standards. Fine aggregate fraction was a mixture of washed sand (0/4 mm) and crushed sand (0/3.15 mm) in order to provide sufficient grading, cohesiveness, and workability. To achieve the necessary skeleton and load-bearing capacity, the coarse aggregate was used in the form of GI gravel (5/16 mm) and GII gravel (10/20 mm). The selection of the material and checking of conformity were carried out so as to reduce variation and assure consistency in the mechanical properties of the test specimen. For the fibers/epoxy composite, the hand lay-up (contact molding) was used to make fibers–epoxy laminates at a fiber mass fraction of 33% wt for each fiber type. Before laying up, the mold was coated with a release agent to ease demolding as well as to have a smooth surface. The supplier had instructions on how to catalyze the resin and apply it to the mold; fiber plies were impregnated and put into resin/fabric alternating sequences with strict consolidation to allow wet-out and reduce voids. Once the gel stage had taken place, more plies were applied to construct the laminate with the desired thickness, which was then allowed to cure in the environment. The cured fiber/epoxy laminate was then placed as an outer coating (wrapping/chemisage) on the concrete specimens, which would create a continuous protective and strengthening coating. The jacketing has two purposes; it offers environmental protection to the substrate by minimizing moisture and chemical ingress, and it strengthens the structure by giving tensile strength and crack-bridging ability along the tensile face during flexure.

### 2.2. Description of Composites (GFRC, JFRC, HFRC)

**Figure 2:**
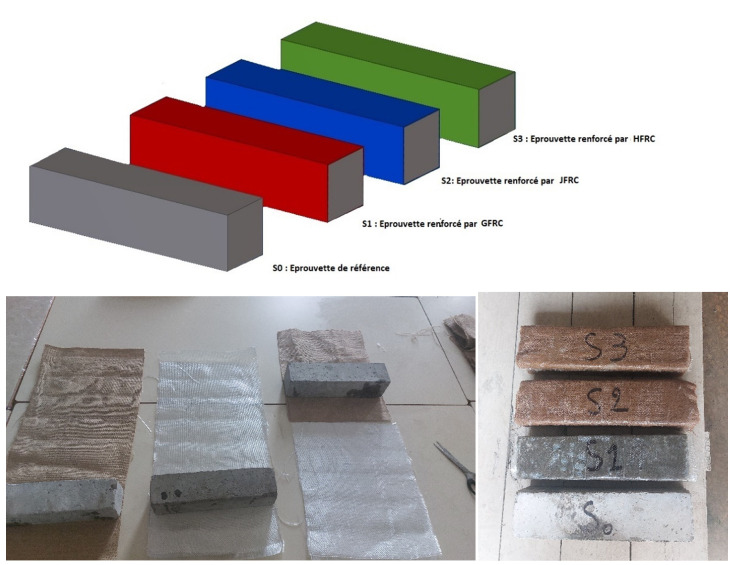
Specimen geometric details.

Twelve concrete specimens were cast in a rectangular shape and made out of normal aggregate concrete with a compressive strength of Pa. The specimens were prismatically cross-sectioned and 280 mm long. The external reinforcement of each specimen was achieved by applying a combination of two layers of composite material as explained above. For specimen S1 made of GFRC (glass fiber-reinforced composite), the composite is composed of two layers of glass fiber fabric, which are usually characterized as having high tensile strength, low elongation, and durability. The epoxy resin was used in wet lay-up in the glass fabric and applied on both sides of the beam. For specimen S2 obtained from JFRC (jute fiber-reinforced composite), the composite is made out of natural jute fiber fabric, which is biodegradable, cheap, and possesses moderate tensile properties. The two layers of jute fabric were applied in the same way as in the case of GFRC. For specimen S3 HFRC (hybrid fiber-reinforced composite), a hybrid system that uses a single layer of GFRC and a single layer of JFRC in alternating order was employed. This combination was supposed to exploit the strength of glass fibers and the sustainability of jute fibers, while also obtaining moderate cost and balanced mechanical properties. The reinforcement operation was conducted as a manual operation with resin impregnation and full-surface wrapping, which provided good fiber-to-matrix bonding and good distribution of the layers. The samples were tested after curing at room temperature for 7 days. In total, we prepared twelve specimens, divided as follows: three specimens for each group (S0, S1, S2, and S3). Table 1 shows a full description and designation of the specimens tested. All the surfaces of the concrete specimens were jacketed with the composite layers. Figure 2 demonstrates an overall picture of both the dimensions of the reference specimen and the composite-reinforced specimens.

For the glass fibers, the typical properties are a tensile strength of 2000 MPa, a Young’s modulus of 72.4 GPa, and an elongation at break of 4.8%. For the jute fiber properties, the tensile strength is 393 MPa, the Young’s modulus is 26.5 GPa, and the elongation at break is 1.5%. For the cured epoxy matrix, the properties values are a tensile strength of 85 MPa, Young’s modulus of 3.5 GPa, and elongation at break of 2.0%.

The composite jacket was prepared like a continuous full-perimeter wrap on the four lateral faces of the prismatic beam using the wet lay-up technique, which is guaranteed to provide uniform impregnation and consolidation of the fabric layers. The sheet ends were overlapped by about 70 mm (one face width), which remained constant for all specimens, and the lap joint was placed intentionally at a lateral face away from the primary tensile region to reduce any possible effect of the seam on the measured flexural response. To reduce the effects of stress concentrations at the edges and to promote jacket continuity, the specimens’ corners were rounded slightly before being wrapped, and the same surface preparation procedure was followed for all the strengthened specimens.

Specimen S0 is the reference beam without any external composite jacketing (0 layers); all strengthened specimens (S1–S3) are different from S0 only because of the presence of the externally bonded composite wrap.

### 2.3. Test Setup and Loading Protocol

**Figure 3:**
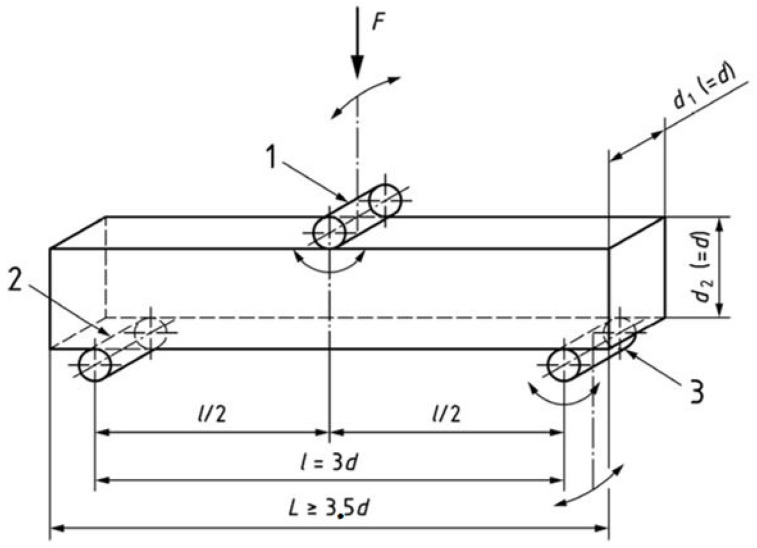
Loading setup of the specimen (three-point loading).

Performances of the reinforced and the non-reinforced concrete beams were determined as per guidelines of EN 12390-5:2019 [21], which defines the test methodology that can be used to assess the flexural strength of hardened concrete. The clear specimen length was based on 210 mm, the span was three times the beam width, which is 70 mm, and the normal geometric proportion was respected during this test. The beams were placed in a symmetric position on two steel support rollers of a diameter equal to 40 mm and a length of 100 mm as shown on Figure 3. All the rollers need to be configured on the positions shown on Figure 3 with a tolerance of 2 mm.

**Figure 4:**
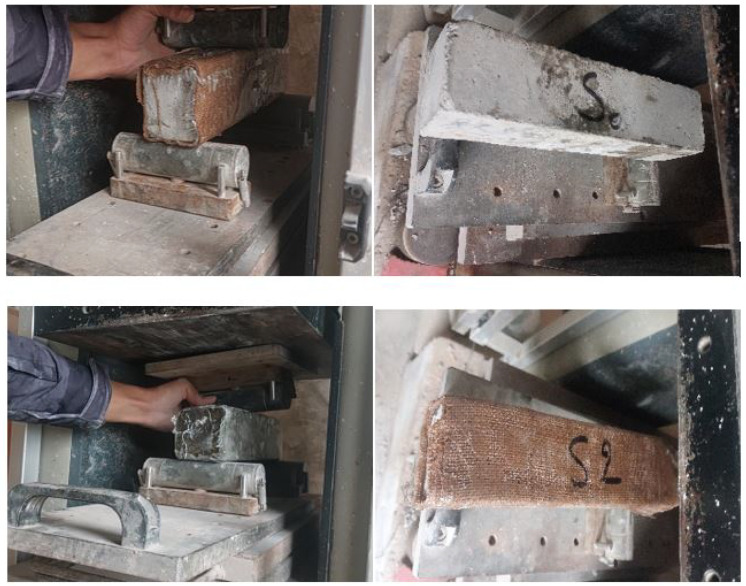
Reinforced and unreinforced beams under flexural loading.

The load was applied in the center using a loading roller with the same diameter. The rollers were all freely rotating and inclinable to ensure the loading process was uniformly applied through stress as shown in Figure 4.

A loading regime with a constant rate of 100 mm/min was used, corresponding to an estimated rate of increase of stress at about 0.05 MPa/s, which may vary between 0.04 MPa/s and 0.06 MPa/s under some conditions. The test was conducted with an imposed displacement of 4 mm for all the specimens, and the load is not applied until the support rollers are properly in contact with the specimen. The mid-span deflection was monitored continuously in order to obtain the complete force–displacement response until 4 mm displacement. At the ultimate load, the specimens were submitted to a visual analysis to take note of typical failure mode, which included the matrix cracking, the fiber–matrix debonding, and the crushing or spalling of the concrete. This method of testing enabled full testing of the flexural capacity, ductility, and energy absorption quality of the eco-composite reinforcement systems.

Due to the stroke/control limitations of the available testing machine, the monotonic quasi-static tests were performed at a constant displacement rate of 100 mm/min and stopped at 4 mm actuator displacement; thus, the rate-dependent effects and prolonged post-peak response beyond this limit were not investigated in the present study.

## 3. Results

### 3.1. Force–Displacement Behavior

**Figure 5:**
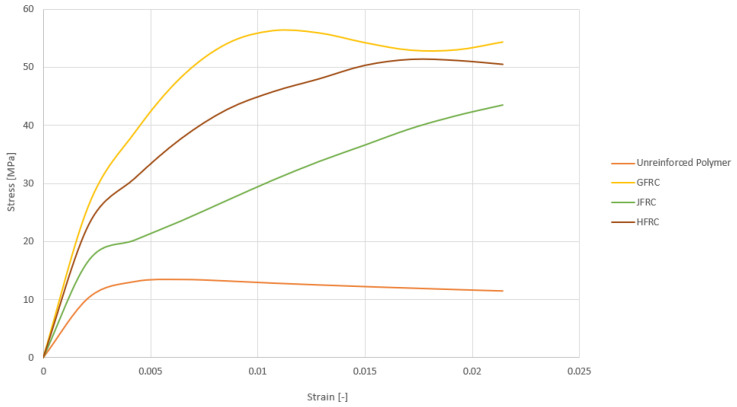
Flexural stress–strain curve.

The stress–strain curves of the tested materials on Figure 5 clearly indicate that there are enormous differences in the mechanical performance between the reinforced and the unreinforced specimens. The control sample S0 for the unreinforced polymer showed standard brittle behavior, with a rather low peak stress of about 13.5 MPa and a strain of just 0.0064, demonstrating poor energy absorption and deformation capacity that result in moderate ductility. By comparison, the GFRC sample recorded a maximum stress of 56.3 MPa, with a strain of 0.0107. This implies significant enhancements in strength and ductility, occasionally accompanied by the incorporation of glass fibers, which offered effective stress transfer and crack-bridging ability under flexural loading. The JFRC structure had a much greater ductility and strength, with the stress plateau reaching a maximum of 43.5 MPa and a strain of 0.0214, which is highest across all materials. Although it has lower flexural strength than the GFRC, the long deformation ability is a sign of successful redistribution of the loads and slow crack growth, as is typical of the natural fiber reinforcement. In the meantime, the HFRC specimen attained a peak strength of 51.4 MPa and retained high strain at failure of 0.0214, which has a similar ductility as that of JFRC but much of the strength of the GFRC was retained. This balanced action indicates synergistic action between glass and jute fibers where stiffness and strength of the synthetic fibers are coupled with greater deformability of natural ones.

The post-yield evolution of stiffness differentiates the three reinforcement systems and is related to the mechanics of the individual fibers in the system. In the GFRC specimen (S1), it is found that the response after the initial cracking is a clear hardening branch, which may be attributed to the rapid transfer of the load from the cracked matrix to the glass fibers with high modulus and stiffness. After that, there is a slight softening stage as micro-cracking and interfacial shear damage accumulate. The hybrid system (S3) forms an extended quasi-plateau with easy softening, which indicates staged damage progression: the glass component maintains a high level of stress, and the jute component provides frictional pull-out and crack-bridging, which moderates the stiffness decay and slows the decay of instability. Nevertheless, the suggested sequence of damage mechanisms (e.g., matrix micro-cracking followed by crack-bridging effects) is based on macroscopic indicators (e.g., shape of the force–displacement curve (post-peak plateau and progressive load reduction) and post-test visual inspection). Since this study did not use acoustic emission, digital image correlation or microstructural/fractographic analysis, these explanations should be treated as qualitative explanations and not as direct experimental confirmation of the underlying failure processes. Future work is also required using Acoustic Emission/Digital Image Correlation and detailed post-morterm analysis, in order to identify crack initiation/propagation paths and validate the proposed staged damage progression. The JFRC specimen (S2) has a continuous and almost monotonic hardening effect until failure, which indicates progressive interaction between the jute network and inelastic deformation, accommodated by interfacial friction and fiber pull-out instead of sudden matrix degradation. Taken together, the qualitative ranking of post-yield stiffness behavior—that is, hardening with small softening in S1, long plateau with mild softening in S3, and continuous hardening in S2—agrees with the expected roles of the fibers, i.e., glass for early stiffness and peak resistance, jute for prolonging the deformation capacity via dissipative mechanisms, and the hybrid architecture for combining these effects to maintain a high stress while moderating the rate of stiffness loss.

### 3.2. Flexural Capacity and Gain (%)

**Figure 6:**
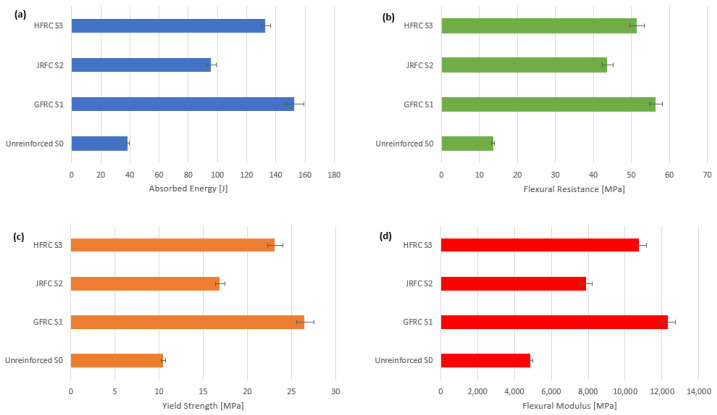
Mechanical performance comparison of S0, GFRC (S1), JFRC (S2), and HFRC (S3): (**a**) Absorbed Energy, (**b**) Flexural Resistance, (**c**) Yield Strength, (**d**) Flexural Modulus (different units).

Figure 6 shows bar charts that clearly give a visual comparison of the mechanical performance of the various composite configurations—unreinforced (S0), GFRC (S1), JFRC (S2) and HFRC (S3)—across four key performance parameters, namely the absorbed energy, flexural resistance, yield strength, and flexural modulus. In this respect, GFRC (S1) shows the greatest values of flexural resistance of 13.51 MPa and yield strength of 26.4 MPa, which means that this material has the best load-carrying capacity and stiffness with respect to flexural stress. These qualities render it especially well-suited to structural components that need high stiffness and load bearing. But HFRC (S3) turns out to be the most moderate performer, with the second-best values in all categories, as well as the highest score of absorbed energy with 132.8 J. This indicates that the material will have a good balance between strength and ductility, and this makes HFRC the most energy-efficient and durable composite under dynamic or impact loading conditions. The fact that HFRC can achieve high flexural resistance and energy absorption indicates the synergistic effect of hybridization, since the addition of jute increases ductility without compromising the stiffness that is imparted by the glass fibers. Although JFRC (S2) does not surpass the configurations based on the synthetic, it provides moderate enhancements in all measures compared to the unreinforced control. Its much greater energy absorption capabilities and flexural strength than those of S0 confirm the promise of natural fiber reinforcement, particularly when sustainability, affordability, and intermediate mechanical properties are sought.

The quantitative analysis, which is summarized in Table 2, indicates that there are important mechanical improvements due to fiber reinforcement of polymer concrete composites. The lowest values of all the parameters measured were recorded in the unreinforced specimen (S0), which had flexural resistance of 13.51 MPa, yield strength of 10.44 MPa, and flexural modulus of 4871 MPa, and can be used as the reference point in terms of performance evaluation. The greatest changes were from the introduction of glass fiber reinforcement (GFRC, S1), with an increase of 297% in absorbed energy, 316% in flexural resistance, and 153% in both the yield strength and flexural modulus. These values show the great stiffness, strength, and energy absorption properties of synthetic fiber systems in flexural stress. Conversely, the jute fiber-reinforced specimen (JFRC, S2) only showed more moderate improvements: 148% in absorbed energy, 222% in flexural resistance, 61% in yield strength, and 61% in modulus. These findings affirm the position of natural fibers in increasing ductility and energy dissipation, as well as providing an alternative that is more sustainable, albeit at the expense of stiffness and strength that synthetic reinforcements present. The hybrid composite (HFRC, S3), which had a combination of both glass and jute fibers, made a strong compromise, as it obtained a 245% increase in absorbed energy, 280% in flexural resistance, and 121% in yield strength and modulus. This balanced performance indicates that the hybridization can synergistically take the benefits of both types of fibers: strength and stiffness of glass fibers and ductility and crack-bridging capability of jute. In general, all reinforced systems performed better than the unreinforced concreate in all parameters, with GFRC performing best in all strength-related metrics, JFRC providing the best ductility, and HFRC performing the best in all metrics. This result highlights the prospect of the fiber-reinforced eco-composites in structural applications that demand high strength and toughness.

As shown in Table 3, the energy-efficiency indicators distinguish the systems according to their effectiveness in energy absorption with regards to the strength measurements. In the case of the energy-to-yield index values, all reinforced specimens display the same high values (GFRC = 5.78; HFRC = 5.76; JFRC = 5.66) as compared to those of the control, S0 = 3.68, implying consistently higher energy uptake at the onset of yielding. In contrast, the energy-to-strength index provides a different ranking where the energy for the control, S0 = 2.85, is higher than GFRC = 2.71, HFRC = 2.58, and JFRC = 2.19, which indicates that at peak load, energy normalization occurred in GFRC and HFRC more often than in JFRC. Combined, the indices indicate convergence of the reinforced systems under the influence of the normalization of yield and divergence under the influence of ultimate strength.

### 3.3. Failure Mode Analysis

**Figure 7:**
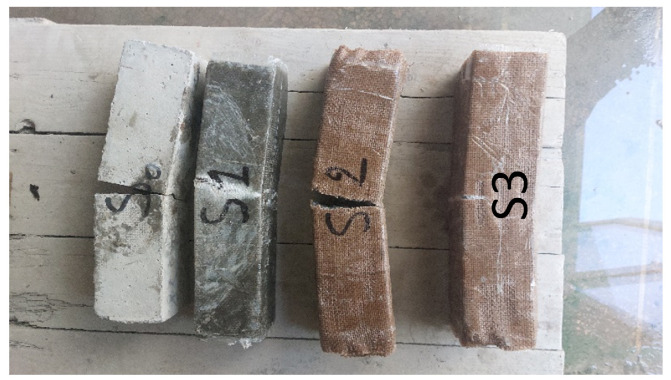
Flexural failure modes of unreinforced and fiber-reinforced concrete specimens: S0 (unreinforced), S1 (GFRC), S2 (JFRC), and S3 (HFRC).

The observation of the cracked specimens on Figure 7 has shown that the earliest and most sudden initiation of cracks takes place in the unreinforced specimen, which is indicative of low tensile strength of cementitious matrix, as well as the lack of a bridging mechanism that could stop the formation of incipient cracks. Reinforced beams, on the contrary, have redirected and bridged crack paths, and mid-span cracking that does not involve apparent delamination of the surface jacket is developed, so the resin–concrete interface can be said to showcase effective mechanical performance during loading. These differences are determined by the nature of the matrix–fiber interaction. Within the glass–fiber system, rapid mobilization of the fibers occurs following the cracking of the matrix through high interfacial shear transfer in addition to raising the initial tangent stiffness and sustaining high peak stresses. Frictional pull-out and progressive fiber engagement are dominant in the system of jute fibers, which results in more smooth post-yield evolution and tolerance to crack opening. The hybrid system involves these mechanisms in a stepwise sequence where the matrix micro-cracking is followed by fiber bridging and a progressive softening faction, which is in line with the high absorbed energy at a strength level close to the glass–fiber system. Taken together, these observations indicate successful bond formation in all reinforcement designs and unique micromechanical mechanisms to improve toughness that are governed by fiber type and hybrid structure. Although no delamination and peeling was observed during testing, this observation alone is not a quantitative assessment of the interface performance. The fact that no separation was apparent under the applied loading range does not rule out that local interfacial damage, partial debonding, or micro-cracking occurred that cannot be identified visually at the specimen scale. Moreover, the precise failure locus (cohesive failure within concrete, adhesive failure at the interface or failure within the laminate) cannot be concluded by visual inspection only. A final evaluation of the quality of the interfacial bond would require a dedicated characterization like pull-off testing and/or microscopic evaluation of the fracture surface. Tensile stresses at the soffit, caused by bending less than three points, are transferred between the cracked concrete and the external composite by the epoxy interface; the quality of this bond determines the level and speed of load sharing. The increased fiber modulus in the glass–fiber system facilitates quick mobilization of the fiber tension once the matrix cracks, which increases the tangent stiffness and gives the increased peak resistance in the GFRC specimen. The modulus of the lower fibers in the jute fiber system, more rugae on the surfaces, and higher pull-out work favors interfacial friction and progressive fiber engagement of the cracks as they open, providing a smoother post-yield evolution and higher deformation capacity as observed in the JFRC specimen. The complete wrapping (chemisage) used in this case aligns the fibers to the beam axis, ensures continuous confinement and anchorage, and is in line with the fact that no beam has peeled or delaminated under all reinforced beams. Mechanistically, such features justify the measured rankings: to be strongest, the glass-dominated GFRC is superior to the hybrid and jute systems; to be most damage-tolerant and energy-dissipative, the hybrid arrangement beats the glass system by giving up the same kind of post-yield strength with the less damaging jute system, with both reinforced systems exceeding the unreinforced control. Table 4 summarizes the failure mode and gain in force according to each configuration. The results of the present section should be interpreted with the view of the scope and instrumentation of the experimental program. The mechanical comparisons are based on global response metrics (load–displacement behavior, calculated stress/strain, and absorbed energy), with no direct evidence of internal damage evolution and interfacial failure modes were collected (i.e., AE/DIC/SEM or pull-off tests). Therefore, any micromechanistic interpretation given in the discussion is meant to uphold the building of the trends and is still inference-based. In addition, variability associated with material heterogeneity and fabrication processes may affect the response; hence, the results reported for trends reflect the specimens tested and the loading configuration. Additional experimental campaigns with extended diagnostics and bond characterizations are suggested in order to reinforce the conclusions of the mechanistic aspects as well as to improve the generalizability.

### 3.4. Material Sustainability Indicators

#### 3.4.1. Energetical Indicators

Table 5 summarizes the values of absorbed energy, yield/ultimate stresses, modulus, and geometry-normalized values. All jackets significantly enhance strength (via) as compared to unreinforced control (S0). GFRC (S1) is the best with an energy density equal to 111,156 J/m^3^, an energy per area of 1945 J/m^2^, and a high ductility index = 2.131. HFRC (S3) offers an equalized enhancement of the energy density equal to 96,790 J/m^3^, a slightly inferior energy per area equal to 1693 J/m^2^ with a close value to S1 of =2.229, and a lower synthetic content. JFRC (S2) has the greatest ductility index = 2.584, with significant increases in toughness equal to 69,477 J/m^3^ and reduced peak stress compared with S1 and S3, in line with less roughness observed in post-yield behavior due to fiber pull-out and bridging.

In this section, the flexural strain reported is an equivalent (nominal) strain based on global response assumptions in the beam and is used as a comparative indicator between configurations, rather than being a direct measurement of a local measurement of strain in the material. Since the instrumentation that is provided mainly offers load and mid-span displacement, without surface strain gauges or complete field strain measurements, the strain calculation is based on a simplified formulation that is commonly used for comparing specimens tested under identical geometry and loading conditions. As shown in Figure 5, the response is nonlinear after cracking. Thus, the reported strain values should be interpreted as an index of global deformation and not as an indicator of linear elastic behavior to the point of yield or ultimate stress. A more rigorous characterization would need to be based on direct strain/curvature measurements (e.g., strain gauges or digital image correlation) and is recommended for future work.

This limitation is expected to be more influential for configurations with a higher post-cracking nonlinearity (e.g., JFRC), for which the nominal strain may under- or overpredict the actual deformation capacity at certain levels of stress. Nevertheless, because the specimens are subject to the same span, cross-section, and loading configuration, the same nominal strain definition that is adopted here offers a consistent basis for relative comparison, even though it does not represent local nonlinear strain fields.

#### 3.4.2. Toughness per Mass (TPM)

Mass and efficiency computations are taken in full-wrap (chemisage) configuration. Beam geometry is set to b = h = 70 mm and L = 280 mm, giving $A_{\mathrm{wrap}}=2\left(b+h\right)L=0.0784$ m^2^. The composite laminate is known as a two-phase system (fibers + epoxy) that is made through hand lay-up of fiber in a fiber mass fraction of Wf = 0.33% wt and is applied across the entire perimeter. Estimates of jacket mass based on nominal laminate densities are related to this Wf and are determined using mass–volume conversion based on constituent densities; unless specified to the contrary, we take GFRC $\rho_{\mathrm{lam}}=1277$ kg/m^3^, JFRC $\rho_{\mathrm{lam}}=1212$ kg/m^3^, and HFRC $\rho_{\mathrm{lam}}=1242$ kg/m^3^. Thickness invoked is in millimeters and expressed as 1 mm. These assumptions would be uniform fiber wet-out, no voids, maximum adhesion to the substrate, and constant properties across the span; any deviation such as varying mass fraction of fiber, incomplete coverage, overlaps, or different constituent densities should be negligible.

Table 6 shows the mass breakdown that will be used for toughness per synthetic mass. Since we used 33% wt of fiber in the woven structure, the mass of fiber will be mf = 0.33*mj and the mass of epoxy will be mepoxy = 0.67*mj. The mass of glass in GFRC is structurally similar to the mass of fiber in JFRC. However, the mass of fibers in HFRC follows the 50/50 rule.

TPM in Table 7 measures the amount of energy [J] each kilo of jacket puts into the structure. Using the full-wrap geometry $A_{\mathrm{wrap}}=0.0784$ m^2^ and nominal laminate densities, the GFRC (S1) has the largest TPM equal to 1523 J/kg, which is the highest toughness return per mass added. The HFRC (S3) has a performance and a small mass penalty of 1364 J/kg, and the JFRC (S2) has a lower TPM of 1003 J/kg as the peak loads and energy absorption are lower than S1 and S3. These trends are indicative of hierarchies in material stiffness/strength in terms of jacket mass alone.

#### 3.4.3. Toughness per Synthetic Mass (TSM)

TSM separates toughness as provided on Table 8 for each kilogram of synthetic material (glass + epoxy), not including jute as a biogenic. HFRC (S3) has the highest TSM equal to 1641 J/kg synthetic, which means that synthetic material is used the most to produce energy absorption. GFRC (S1) surpasses it with 1525 J/kg synthetic; however, JFRC (S2) is almost similar to GFRC (S1), although it has a less prominent peak due to its smaller synthetic mass.

#### 3.4.4. Stored Biogenic CO_2_

A representative level of biogenic CO_2_ in the jute mass in the jacket of 45% has permitted the calculation of the biogenic CO_2_, as reported in Table 9. The JFRC (S2) comprises approximately 0.052 kg of CO_2_, the HFRC (S3) comprises approximately 0.026 kg of CO_2_, while GFRC (S1) has none. These values emphasize the biogenic component of hybrid/natural solutions. From the literature, we have the dry jute ≈45% carbon by mass. $m_{C}$ = 0.45 × mjute We convert carbon to CO_2_ using molar mass ratio 44/12 = 3.667. CO_2 Bio_ = 3.667 × $m_{C}$ = 3.667 × 0.45 × $m_{\mathrm{jute}}$, so the combined factor is 3.667 × 0.45 = 1.65015 × $m_{\mathrm{jute}}$. We use a dry, uncontaminated jute; moisture or resin content would change the effective carbon fraction.

Accordingly, the presented sustainability results should be interpreted not as a full life cycle assessment but as first-order, indicative trends, and the presented environmental benefits should be verified in future work by embodied energy and Global Warming Potential (GWP) analysis.

## 4. Discussion

### 4.1. Global Force–Displacement Response

The force–displacement responses exhibit an observable change in successively damage-tolerant behavior of the beams when they are jacketed. The unreinforced control (S0) peaks at one point and collapses suddenly, which symbolizes the low tensile strength in flexure of plain concrete and the lack of bridging after soffit cracks. The jackets of all the beams (S1–S3) exhibit a steeper initial slope, greater peak load, and broadening post-peak. This development suggests prior loading across the tensile surface to the laminate and a more diffuse damage mechanism following initial cracking, permitting greater deflection mid-span before capacity loss. Similar studies have focused on the same configuration. Unreinforced controls tend to peak and crunch once, whereas jacketing always enhances the force–displacement response of RC beams by steepening the initial slope, increasing the peak load, and extending the after-peak branch, which are characteristics of higher damage tolerance and ductility [23,24,25]. Recovery and even higher original stiffness and strength can be achieved through section enlargement, although brittle debonding can lead to load reductions in certain experiments [23]. In configurations, jacketed beams experience a greater level of deformation capacity and an equivalent amount of shear capacity as specimens without shear reinforcement, with large magnifications [26]. Jacketing also changes brittle shear into ductile flexure; SRG systems doubled peak load by an average of 2835, displacement ductility by an average of 912, and full wraps doubled or tripled strength and displacement ductility (usually by tensile rupture of cords) [24]. Configuration is a factor of efficiency: U-wraps are less likely to suffer shear due to low density, whereas high-density fabrics fail to penetrate mortar [24]. Thickness is important, which increases peak/residual displacements by up to 68% in the presence of the blast, but with an increased risk of sudden failure at very high demand [27]. The materials used have been shown to affect the shape of the curve: increased fiber volume fractions increase the slope at the beginning and control changes in damage, whereas pre-damage steel-fiber jacketing enhances ultimate capacity and displacement compared to post-damage jacketing [28,29]. Last but not least, the quality of the interface is crucial: the enhancement of bond and flexural resistance is caused by surface trimming/roughening, the inhibition of slip and the domination of monolithic action by a robust interface, and the encouragement of brittle debonding by weak bonding [23,30].

### 4.2. Flexural Capacity and Elastic Stiffness

The gain in strength and rigidness is eminent. Flexural peak is the lowest in the control specimen (S0) and the highest in GFRC (S1) and HFRC (S3). The same can be said for yield stress, which demonstrates prior and more intensive interactions between the jacket and systems that contain synthetics. The first linear segment results in the elastic flexural modulus are more important for GFRC (S1) and less important for HFRC (S3), JFRC (S2), and the unreinforced control (S0); this proves that jacketing is not only increasing capacity but is also making the section stiffer before cracking. Stress is denoted as (Unit) MPa, but the raw tables represent stress physically as MPa. All interpretations use it as MPa. In recent literature, the addition of fibers or synthetic reinforcements to concrete uniformly increases rigidity and strength—steepening the force–displacement slope at the beginning, increasing the responses of the peak/yield, and enhancing reinforcement engagement—compared to controls with no reinforcement [31,32,33,34]. Increasing stiffness is proportional to superior grade of concrete strength, additional layers or wall thickness of jackets, and circular confinement, which indicates the greater modulus of elasticity of stronger matrices and the effect of confinement [31,32]. Microfibers made of steel, polypropylene, and other materials also increase compressive, tensile, direct shear, and flexural strength, and their improvement is controlled by the type of fiber, fiber length, and fiber volume fraction [33,34]. As a result, flexural strength and rigidity have the lowest control in their controls and the highest in the system of GFRC/HFRC [31,32,33,34]. Serviceability design is based on the interpretation of these gains, by computing tangent, secant (at many times the peak), or chord moduli on the basis of the first linear (elastic) portion of the stress–strain curve; they are used in serviceability design and are normally reported in MPa [27,31,35,36]. Away from the elastic range, the curve switches from a cracking/plasticity phase to a post-peak softening phase, with jacketed or fiber-reinforced components having higher residual capacity than controls, which indicates that jacketing reduces both the pre-cracking stiffness and total load-bearing capacity [27,31].

### 4.3. Ductility Indices in Strain Space

Measures of deformation on strain space bolster the same image. The coefficient of ductility rises to large values of all reinforced beams compared with S0. The strain–space ductility of JFRC (S2) is the highest, although the maximum stress is lower; HFRC (S3) has an intermediate strength approaching that of near-GFRC with deformation enhancement; additional ductility of GFRC (S1) is clearly increased compared to the control, in spite of its higher stiffness. This division of S1—high strength/stiffness, S2—high ductility, and S3—balanced is the foundation of the comparison between the roles of the fiber systems. Across the literature, ductility in RC beams is the ability to create large, inelastic deformations under excess yield while retaining limited loss in load capacity—vital under seismic, wind, and traffic conditions [27,37,38,39,40]. Two families of metrics are dominant: displacement-based indices and energy-based measures, which are appropriate for FRP systems that lack a distinct yield point [41,42,43,44,45]. Empirically, a higher ductility for reinforced beams is also observed in comparison with the control, and material trends are often observed to favor JFRC in strain space ductility, HFRC as a medium, and GFRC above control (despite higher stiffness). FRP-RC beams are typically found to have lower ductility as compared with steel-RC but can be tuned through reinforcement ratio [27,38,44]. Overall, both displacement and energy-based frameworks are all capable of capturing the improved deformation capacity of reinforced beams and provide complementary perspectives for steel- and FRP-reinforced members [43,44,45].

### 4.4. Energy Absorption and Geometry-Normalized Toughness

The region under the force–displacement curve is an indication of absorbed energy, which rises significantly due to jacketing. Division by specimen volume V normalizes the results and shows that the absorbed energy of S1 is the highest, S3 is the second-highest, and S2 is below S3 but far above S0. A complementary normalization, which is the division by the wrapped area **$A_{\mathrm{wrap}}$**, also produces the same hierarchy and is convenient when the efficiency of retrofit on a surface basis is being discussed. Such measures reveal that reinforced beams do not merely go to a larger peak, but rather, they store and dissipate much more energy throughout the recorded history of displacement, which translates into damage tolerance in flexure.

Energy absorption in structural and composite specimens is most often expressed in terms of energy absorption during deformation, which is computed as the area under the force–displacement curve, where force can be the applied load and displacement can be the corresponding deformation; this type of definition is applied in both static and dynamic cases like crushing, impact, and cyclic loading [28,46]. In this framework, structural modifications like trigger mechanisms, jacketing, and retrofitting are used for enhancing the absorbed energy by facilitating progressive deformation/crushing and increasing the force–displacement area, and it has been reported that in retrofit specimens, the cumulative absorbed energy is substantially higher than in reference specimens—up to several-fold compared to reference specimens—demonstrating the effectiveness of confinement and retrofit strategies in the enhancement of energy uptake [47]. Because absorbed energy is proportional to the size and mass of the specimen, studies often normalize it by volume or mass, which leads to assessment of specific energy absorption (SEA), which allows the fair comparison of the energy absorption efficiency of different geometrical shapes—with differing volumes and masses—and helps to put different alternatives into a meaningful ranking according to the level of normalized energy absorption performance [48]. Beyond the total area, the shape of the curve provides some diagnostic information: plateaus and progressive stages of deformation generally mean high levels of energy absorption, and there is evidence of a large proportion of energy that can be dissipated during the plateau phase, meaning boundary conditions and failure modes can change the magnitude and location of energy dissipation [49].

### 4.5. Post-Yield Stiffness Character from Stress–Strain Curves

Being the first to crack, GFRC (S1) has a definite hardening curve with a high peak upon softening, in agreement with effective early load transfer to a high-modulus fiber network with only progressive interfacial degradation. HFRC (S3) exhibits a long quasi-plateau with weak softening, which implies progressive damage, that is, matrix micro-cracking, fiber bridging, and gradual reduction in the load as more interfaces come into action. A smoother and almost monotonic increase until failure, as indicated by JFRC (S2), is characteristic of frictional sliding and fiber pull-out as the main dissipation mechanisms. Concisely, glass moderates premature stiffness and peak stress, jute prolongs the deformation by the work-intensive pull-out tendency, and the hybrid is an established balance between these two, restraining softening at higher stress levels. GFRC and HFRC generally exhibit different post-cracking behavior in stress–strain/load–displacement: GFRC usually has a significant strain-hardening branch following initial cracking that attains a high peak and subsequently enters a post-peak softening phase, while HFRC usually displays a longer quasi-plateau with reduced post-cracking softening, which is associated with progressive damage mechanisms that are controlled by mechanisms of micro-cracking, fiber bridging, and gradual interfacial engagement [50]. A similar progressive nature is also exhibited in HFRC under compression with the existence of nonlinear unloading–reloading paths as well as residual strains that are controlled by a damage factor, suggesting a distributed damage evolution process instead of a catastrophic failure [51]. Similar “elastic-hardening–softening” patterns are reported in other fiber-reinforced/high-strength concretes (HSFRC/HSC), supporting the generality of post-peak strain-softening following peak stress [52], but SFRC and PFRC show that post-cracking responses can be considerably different like stress drop and re-hardening for PFRC, although hardening/softening behavior is a common organizing framework for post-cracking interpretation [53]. In parallel, energy dissipation in fiber-reinforced composites is attributed widely to interfacial processes such as matrix cracking, followed by debonding, frictional sliding, and pull-out [or rupture] of the fiber that gives rise to smoother, as well as more monotonic load increases until failure when pull-out and friction are dominating the response [54,55]. The efficiency of these mechanisms highly depends on fiber type and interface properties: stiffer fibers such as glass can increase the initial stiffness and peak stress compared to soft fibers such as jute-like natural fibers, which are more “pull-out prone,” can increase the time of deformation, and increase the toughness; thus, fiber hybridization (“mixing” complementary roles of the fibers) is used to balance stiffness and ductility [56,57]. Load–displacement/force–slip responses are often interpreted in terms of staged interface response—initial elastic adhesion, transition to frictional sliding and potential mechanical anchorage—relating curve shape directly to the underlying pull-out/friction processes controlling toughness and energy absorption; it is also emphasized that excessive debonding and lack of friction can impair load transfer and reduce strength, notwithstanding increased deformation capability [58,59,60,61].

### 4.6. Energy–Strength Composite Indices

There are two small ratios that can be used to explain the scaling of dissipation with strength. The value of the Energy-to-Yield Index is large in S1, S2, and S3—all larger than S0—indicating that at the beginning of yielding, the jackets allow energy uptake to increase significantly compared to the control. The highest of the Energy-to-Strength Index is S0, bridging the variation between the reinforced systems. The Energy-to-Strength Index is smaller for GFRC (S1) and HFRC (S3) since they receive significantly higher flexural strength. Combined, yield-normalized measures focus on increased first-yield dissipation of all jackets, and ultimately, normalized measures show the great benefit of high peak-load of the synthetic-containing systems. Additionally, energy–strength composite indices are also extensively used to compare the efficiency of different materials and reinforced systems in dissipating or absorbing energy with regard to their strength. And from the literature, it is known that dissipation does not follow a linear line with respect to strength unless some kind of normalization is used. Under an impact-type loading with a fixed energy input, the percent of energy dissipated generally diminishes with increasing yield strength (since a larger amount of energy is needed to initiate yielding) and increases with higher Young’s modulus (stiffer materials yield at lower strains), and when results are normalized, dissipation curves from various materials may collapse upon a common trend, which is consistent with scaling laws for predicting dissipation behavior in elastic—perfectly plastic solids, showing weak dependence on the mass of the impactor after normalization [62]. To make these ideas tangible, a number of “dissipation ratios” are defined according to the application: ratios may be given in the form of the fraction of the total dissipation occurring in a certain region/component, as the ratio of dissipated to input energy density during loading-deformation-failure (evolution of their material parameters being characteristic of strain-dependence and their mid-value, meaning that elastic and dissipative contributions are comparable) [63], as area-based ratios that are derived from parts of the load–displacement loops quantify the energy dissipation capacity. A common trend among structural applications is that dissipation indices increase dramatically at the onset of yielding so the yielding devices/jackets exhibit higher energy uptakes than controls after the onset of plasticity, which makes dissipation indices useful in identifying the onset of yielding and in tracking the progress of damage [64,65]. For reinforced masonry/concrete systems, monotonic trends of energy-to-strength indices (and related strength/displacement/energy ratios) might lack straightforward monotonic characteristics with reinforcement index between orientations, which reveals system dependence and encourages the sensitive interpretation of “efficiency” metrics [66]. In strain-hardening cementitious composites, paired indices are often taken—the strength index, which is often associated with the margin between first cracking and ultimate tensile strength, and the tendency of multiple cracking and energy index, which is associated with the complementary energy versus crack-tip toughness and the ability to continue steady-state cracking—with stable strain-hardening [67,68]. Despite their usefulness, the relationship between strength and energy indices is not strictly deterministic—ultimate tensile strain can depend both on these indices and can change with age, and “non-saturated” vs. “saturated” cracking conditions can result in very different crack width patterns at similar index levels [69,70,71,72]. Finally, database-level comparisons are more about uncertainty since energy-related models tend to be more scattered than strength models, because energy is a combination of both force and displacement and because reinforcement-system datasets with limited samples need caution; however, some studies have been reported to have broadly linear strength index correlations with compressive strength across systems, while energy indices and strain capacity tend to increase with age, albeit with outliers [69,70,73,74,75].

### 4.7. Mass-Normalized Performance (TPM) and Synthetic-Content Efficiency (TSM)

Mechanics are related to efficiency by means of mass normalization. The TPM of GFRC is the largest, followed by HFRC, and finally JFRC, which is in the same order as that of strength and stiffness when normalized by added mass. TSM, toughness per synthetic mass composed of epoxy and glass, puts a slight twist on the ranking: the HFRC is the most efficient in converting synthetic material into absorbed energy, with GFRC and JFRC not that far below, since its synthetic content is only epoxy and fibers are biogenic. TPM provides return per kilogram added whereas TSM provides return per kilogram of non-biogenic content, both of which are applicable within a sustainability frame. Mass-normalized performance metrics (TPM) and efficiency metrics based on synthetic contents (TSM) are generally used to compare reinforced concretes, taking into account the added mass and amount of synthetic content, respectively. In general, GFRC has high compressive and flexural performance related to strong resin glass bonding and fairly high elastic modulus [76]. HFRC exhibits wide mechanical advantages over plain concrete in various types of strength modes: normalized ratios are approximately 0.95–1.22 times higher in compressive mode, 1.02–2.16 times higher in direct shear mode, 1.07–2.0 times higher in splitting tensile mode, 1.06–1.42 times higher in four-point flexure mode, and 1.07–1.87 times higher in uniaxial tensile mode [77,78]. By contrast, JFRC may show a decrease in compressive strength when compared to plain concrete, with reported decreases ranging from ~6% to ~22.7%, but they can provide a significantly higher strain capacity at peak load of around +401.5% and substantially higher energy absorption from +43.9% to +61.2%, along with pronounced increases in toughness indices such as +86% flexural toughness and +124% compressive toughness [79,80,81]. When the strength and stiffness are normalized by adding mass (TPM), the general hierarchical order is mostly reported as GFRC, HFRC, and then JFRC [76,77,80]. However, when we consider efficiency in terms of toughness per unit of synthetic mass (TSM), HFRC can be the most efficient in converting the synthetic content into absorbed energy/toughness, including reported toughness values of ~0.64 in compression and ~0.48 in tension, more than natural fiber systems in the same reported data, and sometimes more efficient than GFRC on an efficiency basis even if GFRC is superior in terms of pure mechanical properties [76,77,78]. JFRC can also be seen as relatively efficient in TSM terms because it gives high toughness/energy gains with relatively low synthetic contents, mainly epoxy and biogenic fibers, even if the absolute strength is lower than GFRC/HFRC. Overall, hybridization effects and fiber/matrix parameters are the main drivers of the above-mentioned trade-offs between peak strength, stiffness, ductility, and energy absorption [77,78,79,80,81].

In present study, the flexural resistance increased +316%, +222%, +280% (GFRC, JFRC and HFRC) compared to the unreinforced beams, whereas the absorbed energy increased +297%, +148% and +245% (GFRC, JFRC and HFRC). These gains can be benchmarked with the existing literature about externally bonded FRP and bio-based FRP strengthening of RC members. For example, GFRP laminate/sheet-strengthening systems have been experimentally proven to improve the flexural response of RC beams, with improvements being strongly controlled by bond/anchorage details and laminate configuration [82]. Hybrid natural/synthetic strengthening concepts have been reported for RC beams as well [e.g., hybrid systems with jute and other fibers], with a demonstration of increased flexural performance with lower target synthetic content [83]. More broadly, in a recent comprehensive review on natural fibers and biopolymers in FRP composites for strengthening concrete structures, it is pointed out that bio-based FRPs can effectively improve load capacity and energy-related performance, but it points out the sensitivity of the results to quality of manufacture, interface bonding, and durability [84]. In addition, several cementitious-matrix alternatives have been reported in the literature as potential retrofitting solutions for flexural strengthening of RC beams, which include textile-reinforced mortar (TRM). They could be used as a benchmark class to compare the strength–ductility trade-offs [85]. Within this context, the current TPM/TSM assessment has set the place-proposed hybrid wrap as a competitive answer when the efficiency is normalized in extra mass and synthetic content, while recognizing that strict one-to-one comparisons throughout studies are affected by geometry, strengthening length, quantity of layers, and test set-up.

### 4.8. Failure Modes and Interface Quality

The inferred mechanisms are confirmed by observations of failures. All reinforced beams collapse by crushing through flexural cracking in the absence of reinforcement delamination; the control collapses in a brittle way once it has broken its first crack. Lack of peeling is a sign of a good bond between the epoxy and concrete in the full-wrap system, allowing the stress to be spread uniformly across the tensile face and bridging cracks. This interface performance is the reason why there are high peak strengths as well as widened post-peak branches in the reinforced systems, with the much longer, damage-resistant responses of the jute and hybrid jackets in the displacement range, in particular, being tested. Reinforced concrete beams reinforced by epoxy-bonded external reinforcement such CFRP or steel plates typically experience flexural cracking, and if the adhesive bond is sufficiently strong, they develop desirable “full-utilization” failures like concrete crushing in compression after steel yielding or even FRP rupture due to good stress transfer and crack-bridging delaying premature peeling or debonding [46,86,87,88]. On the other hand, poor bonding, associated with poor surface preparation, inadequate bond length, or insufficient bond reinforcement configuration, can lead to the onset of unfavorable brittle modes of plate peeling/delamination, epoxy–concrete interface debonding, rod pull-out, or concrete cover separation, where the predominant mechanism depends on reinforcement stiffness/area, anchorage, and bond capacity [89,90,91,92,93]. Post-failure evidence is often observed in which well-bonded systems fail in a thin layer of concrete adjacent to the adhesive or pull off a wedge of concrete with the reinforcement, suggesting that the adhesive bond strength can be greater than the tensile capacity of the substrate [94,95,96]. More generally, the post-peak behavior of reinforced systems is strongly controlled by interface behavior and fiber characteristics: the addition of fibers (steel, jute, hybrid) tends to enhance the peak load and the post-peak branch, whereas the longer the fiber length and the higher the volume fraction/aspect ratio and favorable orientation/distribution, the better the crack-bridging, residual strength, CMOD capacity, and damage tolerance appear to be as compared to the only variation of matrix strength [97,98,99], Reinforcement placement can be used to alter the post-peak branch and confinement strategies (stirrups or external jackets such as CFRP) usually increase the deformability and decrease the brittleness, although increases in confinement are not always accompanied by proportional increases in peak strength [100,101,102,103]. Due to the lack of AE/DIC/SEM investigations and bond characterization tests, mechanistic interpretations that do not represent direct experimental confirmation are presented in a plausible manner that is consistent with the macroscopic behavior.

### 4.9. Synthesis for a Sustainability Context

The reinforced beams are more elastically stiff, have significantly greater peak capacity, greater deformation before instability, and absorbed energy significantly greater than the unreinforced control. GFRC optimizes peak measures, JFRC optimizes strain–space ductility and post-yield smooth evolution and HFRC has a robust strength–ductility compromise and has a better material efficiency, normalized by synthetic content. Energy- and strain-based indices reveal that all eco-composite jackets significantly increase toughness when compared to the unreinforced beam, and the hybrid especially is quite an attractive alternative when the structural performance has to be compromised against the material usage, which is eco-friendly. The literature is consistent in showing that the fiber-reinforced and hybrid RC beams, especially the ones with GFRP reinforcement, tend to have higher elastic stiffness, higher peak load capability, and even better energy absorption behavior compared to the control in unreinforced cases, while these benefits could be increased even further when advanced cementitious materials like UHPC or ECC are used, which can improve the cracking load, maximum load, ductility, shear capacity, deformation capacity, and crack control [104,105]. At the same time, the behavior of the deformation of the GFRP-RC system is different from that of the steel-RC system due to the lower elastic modulus of GFRP bars that lead to greater mid-span deflections and stiffness of the structure compared with the steel reinforcement, where the section mechanics, such as the neutral axis closer to the compression zone, contributes to greater deflections and potential lower shear strength than the CFRP- or steel-reinforced alternatives [106] and over-reinforced high- and ultra-high-strength GFRP-R. According to material-selection studies, the results also show that the GFRP plate can be more efficient than the CFRP plate when the design goal is to maximize the stiffness, whereas, for maximizing the ultimate load, the CFRP systems may become the preferable choice, and hybridization strategies, such as the HFRP/HFRC concepts, and fiber geometry optimization, such as hooked steel fibers, often possess a preferable strength–ductility compromise and efficiency normalized by the synthetic content [46,107]. In parallel, eco-composite and hybrid jacketing solutions greatly increase the beam toughness and energy dissipation compared to unreinforced references, with some of the highest reported improvements in the post-peak damage tolerance using synergistic combinations of PP fibers plus steel wire mesh in ECC, as well as significant energy dissipation improvement brought by ECC jacketing, contributing to the general conclusion that composite jackets can greatly increase the post-peak damage tolerance while allowing for more sustainable performance trade off [108].

## 5. Conclusions

This study shows that the full-wrap external jacketing using GFRC (S1), JFRC (S2) and HFRC (S3) considerably improved the three-point bending performance of B30 concrete beams compared with the unreinforced control (S0). All jacketed specimens had higher initial stiffness, higher peak load, and a broader post-peak response, which would indicate better load-carrying ability and deformation tolerance. Compared to S0, the absorbed energy was increased by +297, +148, and +245% (S1, S2, and S3), and the flexural resistance was increased up to +316%, the yield strength was increased up to +153%, and the flexural modulus was increased up to +153%. The failure observations were consistent between the strengthened and control beams, with flexural cracking followed by concrete crushing but no delamination of the concrete, whereas the control beam failed abruptly upon first major cracking. In terms of normalized performance, GFRC showed the highest energy return per unit mass added (TPM = about 1523 J/kg), HFRC had the highest toughness per unit synthetic mass (TSM = about 1641 J/kg), and JFRC had the lowest peak performance but achieved significant energy absorption performance gains and the highest biogenic contribution (about 0.052 kg stored CO_2_ in S2). From a sustainability point of view, the availability of both TPM (toughness per total jacket mass) and TSM (toughness per synthetic mass) offers a useful complementary framework that reflects performance efficiency while also explicitly taking into account the use of non-biogenic constituents (epoxy and glass). In addition, the addition of jute in JFRC/HFRC brings a measurable biogenic fraction (stored CO_2_) in favor of recyclability and circularity-driven strengthening choices for RC rehabilitation in addition to the mechanical improvements noted.

## 6. Limitations and Future Work

The present study has evaluated the short-term flexural response of the proposed strengthened beams under monotonic loading conditions. Consequently, the questions of long-term durability were not assessed. This is especially true for natural fibers such as jute that might be sensitive to moisture uptake, biological degradation, and alkaline environments typical of cementitious materials, potentially affecting their fiber integrity and the fiber-matrix/interface performance over time. Future work will therefore concentrate on durability-related investigations using accelerated aging and environmental conditioning protocols such as wet/dry cycling, water immersion, alkaline exposure, and temperature/humidity conditioning, followed by a residual mechanical test to determine the strength, stiffness, and energy absorption retention.

## Figures and Tables

**Table 1:** Specimens reinforcement types.

Specimen	Layers	Reinforcement Type
S0	0	None (reference beam)
S1	2	Glass Fiber-Reinforced Composite (GFRC)
S2	2	Jute Fiber-Reinforced Composite (JFRC)
S3	2	Hybrid Fiber Composite (1 layer GFRC + 1 layer JFRC)

**Table 2:** Comparative mechanical properties of unreinforced and fiber-reinforced polymer concrete specimens (mean ^+^/^−^ SD, SD <5%).

Specimen	Absorbed Energy [J]	% Gain in Energy	Flexural Resistance [MPa]	% Gain in Flexural Resistance	Yield Strength [MPa]	% Gain in Yield Strength	Flexural Modulus [MPa]	% Gain in Flexural Modulus
Unreinforced S0	$38.44_{-0.97}^{+1.19}$	-	$13.51_{-0.38}^{+0.46}$	-	$10.43_{-0.24}^{+0.29}$	-	$4871.33_{-96.15}^{+118.40}$	-
GFRC S1	$152.50_{-5.75}^{+6.90}$	297%	$56.26_{-1.62}^{+1.95}$	316%	$26.40_{-0.84}^{+1.12}$	153%	$12322.34_{-355.70}^{+410.20}$	153%
JRFC S2	$95.32_{-2.96}^{+3.85}$	148%	$43.54_{-1.28}^{+1.64}$	222%	$16.84_{-0.47}^{+0.61}$	61%	$7862.61_{-285.40}^{+352.10}$	61%
HFRC S3	$132.79_{-2.65}^{+3.42}$	245%	$51.38_{-1.74}^{+2.08}$	280%	$23.05_{-0.79}^{+0.98}$	121%	$10759.57_{-318.20}^{+389.60}$	121%

**Table 3:** Energy-efficiency indices under three-point bending (energy-to-strength and energy-to-yield).

Specimen	Unreinforced S0	GFRC S1	JRFC S2	HFRC S3
Energy-to-Strength Index	2.85	2.71	2.19	2.58
Energy-to-Yield Index	3.68	5.78	5.66	5.76

**Table 4:** Failure mode for and gain in force according to each specimen.

Designation	F (N)	% Gain in F	Failure Mode
S0	10.6	–	Normal concrete crushing: typical brittle cracking
S1	37.8	256%	The surface layer appears well-adhered: No reinforcement delamination, followed by concrete crushing
S2	13.9	31%	Conservation of the surface layers adherence: No reinforcement delamination, followed by concrete crushing
S3	20.6	94%	Well-adhered surface layers: No reinforcement delamination, followed by concrete crushing

**Table 5:** Energetic and deformation indicators from three-point bending.

Specimen	$E_{\mathrm{abs}}$ [J]	$\sigma_{y}$ [MPa]	$\sigma_{u}$ [MPa]	*E* [MPa]	$\varepsilon_{y}=\sigma_{y}/E$	$\varepsilon_{u}=\sigma_{u}/E$	$\mu_{\varepsilon }=\varepsilon_{u}/\varepsilon_{y}$	Energy Density $=E_{\mathrm{abs}}/V$ [J/m^3^]	Energy per Area $=E_{\mathrm{abs}}/A_{\mathrm{wrap}}$ [J/m^2^]
S0 (Unreinforced)	38.443	10.439	13.512	4871.33	0.002143	0.002774	1.294	28,019	490.34
S1 (GFRC)	152.507	26.405	56.265	12,322.34	0.002143	0.004565	2.131	111,156	1945.24
S2 (JFRC)	95.323	16.848	43.541	7862.61	0.002143	0.005538	2.584	69,477	1215.85
S3 (HFRC)	132.796	23.056	51.385	10,759.58	0.002143	0.004776	2.229	96,790	1693.83

**Table 6:** Jacket mass breakdown for recyclability accounting.

Specimen	$m_{j}$ (kg)	$m_{f}=0.33\;m_{j}$ (kg)	$m_{\mathrm{epoxy}}=0.67\;m_{j}$ (kg)	$m_{\mathrm{glass}}$ (kg)	$m_{\mathrm{jute}}$ (kg)
S1 (GFRC)	0.100	0.0330	0.0670	0.0330	0.0000
S2 (JFRC)	0.095	0.0314	0.0636	0.0000	0.0314
S3 (HFRC, 50/50 fiber mass)	0.097	0.0320	0.0650	0.0160	0.0160

**Table 7:** Toughness per mass (TPM) under full-wrap 1 mm jackets.

Specimen	$A_{\mathrm{wrap}}$ (m^2^)	$\rho_{\mathrm{lam}}$ (kg/m^3^)	Thiknesst (mm)	$m_{\mathrm{jacket}}$ (kg)	$E_{\mathrm{abs}}$ (J)	TPM $=E_{\mathrm{abs}}/m_{\mathrm{jacket}}$ (J/kg)
GFRC (S1)	0.0784	1277	1	0.100	152.507	1523.3
JFRC (S2)	0.0784	1212	1	0.095	95.323	1003.2
HFRC (S3)	0.0784	1242	1	0.097	132.796	1363.8

Wrapped area (full wrap): $A_{\mathrm{wrap}}=2\left(b+h\right)L$ [m^2^]; Mass per mm thickness: $M/mm_{\mathrm{of}\;\mathrm{thikness}}$ [kg/mm] = $\rho_{\mathrm{lam}}$ [kg/mm^3^] $\times \;A_{\mathrm{wrap}}$ [m^2^]; Jacket mass: $m_{\mathrm{jacket}}$ [kg] = $M/mm_{\mathrm{of}\;\mathrm{thikness}}\times \;t$; TPM: TPM [J/kg] = $Eabs/m_{\mathrm{jacket}}$.

**Table 8:** Absorbed Energy impact on TSM.

Specimen	$E_{\mathrm{abs}}$ (J)	$m_{\mathrm{syn}}$ (kg)	TSM (J/kg)
S1 (GFRC)	152.507	0.1000	1525.1
S2 (JFRC)	95.323	0.0636	1498.8
S3 (HFRC)	132.796	0.0810	1640.7

$m_{\mathrm{syn}}=m_{\mathrm{epoxy}}+m_{\mathrm{glass}};\mathrm{TSM}=\frac{E_{\mathrm{abs}}}{m_{\mathrm{syn}}}.$

**Table 9:** Stored biogenic CO_2_ from jute content.

Specimen	Jute Mass (kg)	CO_2 Bio_
S1 (GFRC)	0	0
S2 (JFRC)	0.0314	0.052
S3 (HFRC)	0.016	0.026

## Data Availability

Data is contained within the article: The original contributions presented in this study are included in the article. Further inquiries can be directed to the corresponding authors.
